# Update on the medical management of fibrous dysplasia of the bone

**DOI:** 10.1177/20420188251347350

**Published:** 2025-06-18

**Authors:** Kelly L. Wentworth, Jeayoung Park, Xiaobing Yu, Edward C. Hsiao

**Affiliations:** Division of Endocrinology and Metabolism, Department of Medicine, University of California, San Francisco, 513 Parnassus Ave., HSE901G, San Francisco, CA 94143-0794, USA; Endocrine Research Unit, San Francisco Veterans Affairs Health Care System, 1700 Owens Street, Room #368, San Francisco, CA 94158, USA; Division of Endocrinology and Metabolism, Department of Medicine, University of California, San Francisco, San Francisco, CA, USA; Center for Pain Medicine, Division of Pain Medicine, Department of Anesthesia and Perioperative Care, University of California, San Francisco, San Francisco, CA, USA; Division of Endocrinology and Metabolism, Department of Medicine, University of California, San Francisco, 513 Parnassus Ave., HSE901G, San Francisco, CA 94143-0794, USA; Institute for Human Genetics, University of California, San Francisco, 513 Parnassus Ave., HSE901G, San Francisco, CA 94143-0794, USA

**Keywords:** bisphosphonates, fibrous dysplasia, McCune-Albright syndrome, metabolic bone disease, pain management

## Abstract

Fibrous dysplasia (FD) is a rare, benign skeletal disorder characterized by expansile, fibrotic bone lesions that replace normal bone, resulting in decreased bone strength, pain, and fractures. The clinical presentation of FD can vary widely, complicating the diagnosis. FD can manifest as monostotic (single bone) or polyostotic (multiple bones) disease and can occur independently or as part of McCune–Albright Syndrome (MAS), a genetic condition that includes café-au-lait skin hyperpigmentation and endocrine abnormalities. FD/MAS arises from activating mutations in the *GNAS* gene, leading to constitutive activation of the G_s_α protein and elevated cAMP levels. Despite understanding the genetic cause of FD, effective treatments remain limited. Current management strategies focus primarily on symptom control following the most recent comprehensive guidelines published in 2019. This review highlights emerging pharmacologic treatments, including denosumab, a monoclonal antibody that has shown promise in reducing lesion size and pain in FD patients, and burosumab, a monoclonal antibody targeting FGF23, which reduces renal phosphate wasting and osteomalacia in FD patients. In addition, we review updates in advanced genetic testing techniques, such as cell-free DNA and direct lesion sampling for next-generation sequencing, which are promising methods for improving the diagnostic accuracy of FD. Finally, multimodal approaches for pain management in FD, including nonsteroidal anti-inflammatory drugs, bisphosphonates, and novel agents like cannabinoids, are being used alongside the traditional approaches with physical therapy and psychological support. Ongoing research aims to enhance our understanding of FD pathogenesis and develop targeted therapies that could potentially reverse disease progression. This review underscores the importance of implementing a multidisciplinary approach in the management of FD/MAS and finding new therapeutic approaches that will help address the diverse manifestations and improve the quality of life for patients.

## Introduction

Fibrous dysplasia (FD) of the bone is a genetic skeletal dysplasia characterized by the abnormal formation of fibrous tissue in place of normal bone, leading to decreased bone strength, expansile bone lesions, and fractures.^1^ FD accounts for 2.5% of all bone lesions and 7% of benign skeletal dysplasias,^2^ with craniofacial and long bones being major sites.^3^ Although the genetic mutations that lead to FD are known and are located at the *GNAS* locus, medical treatments for this disfiguring disorder are sorely lacking.^4^ FD most commonly occurs in isolation as monostotic disease, but can also affect multiple bones (polyostotic FD) or be part of McCune–Albright Syndrome (MAS), a somatic mosaic genetic condition characterized by polyostotic FD, café-au-lait skin hyperpigmentation, precocious puberty, endocrinopathies (e.g., Cushing’s disease, hyperthyroidism, acromegaly), and solid organ malignancies.^5–7^

FD/MAS is caused by activating mutations in the *GNAS* locus, encoding the G_s_α protein.^4^ The most common variants, c.602G>A (p.R201H) and c.601C>T (p.R201C), cause constitutive activation of G_s_α by inhibiting its GTP hydrolase activity, leading to persistently elevated intracellular cAMP and increased downstream cAMP pathway activity. These mutations occur post-zygotically, resulting in tissue mosaicism, and are not inherited through the germline. As a result of this mosaicism, the clinical disease spectrum of FD ranges from single-bone involvement to multi-organ involvement.

Although MAS affects numerous tissues, FD can develop independently of these other conditions and is arguably the most significant manifestation of MAS due to the lack of pharmacologic treatments for the bone complications.^8^ Medical management for FD has largely been symptomatic and guided by expert opinion in management guidelines published in 2019.^8^ This review discusses some of the major advances in our understanding of FD over the past 5 years and how these findings may impact the medical management of patients with FD.

## Clinical presentation of FD

**Figure 1.**
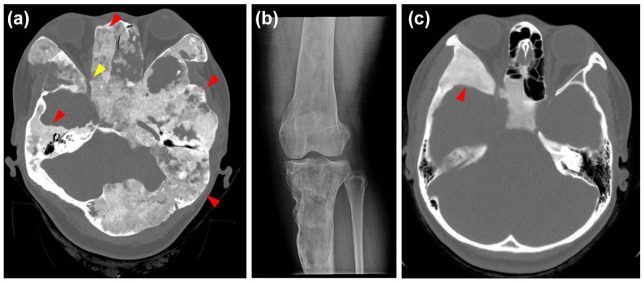
Radiologic imaging of fibrous dysplastic bone. (a) Craniofacial CT scan of a 33-year-old female with FD/MAS, showing extensive fibrous dysplastic bone in the skull (representative lesions, red arrows). Narrowing of the optic canal can be seen in this image (yellow arrow). In addition, this craniofacial lesion shows cystic changes that can be seen in some FD lesions. (b) FD of the right tibia as seen on plain film X-ray, of the same patient at age 29. (c) FD of the craniofacial region in a 16-year-old female, showing the classical ground glass-like lesion. CT, computed tomography; FD, fibrous dysplasia; MAS, McCune-Albright syndrome.

FD occurs across a wide phenotypic spectrum and is a common congenital skeletal dysplasia that can affect one or more bones,^4^ leading to expansile, fibrotic bone lesions that cause fragility, malformations, and pain (Figure 1). FD can also occur in the context of MAS. The broad clinical spectrum of FD/MAS and the mosaic nature of the disease lead to variability in the radiographic presentation, making FD challenging to accurately diagnose. Monostotic FD is thought to be much more common than reported because the lesions can be small, symptoms may be mild, and the lesions may be discovered only on ancillary imaging for other causes, such as headache or after injury.^9,10^

## Initial diagnosis

The WHO classification of soft tissue and bone tumors defines FD as: (1) a bone lesion with compatible imaging characteristics (ground glass trabecular expansion of bone), (2) an osseous portion consisting of irregular curvilinear branching trabeculae of woven bone without apparent osteoblastic rimming on histopathology, and (3) a fibrous portion consisting of bland fibroblasts on histopathology.^11^ The presence of an activating *GNAS* mutation within the bone lesion provides additional confirmatory evidence of FD; however, the diagnosis of FD is frequently made on the basis of radiographic findings, without the need for biopsy. Classic radiographic features of FD include an expansile, well-defined intramedullary lesion with a ground glass appearance, cystic, sclerotic, or mixed cystic–sclerotic features, and cortical thinning with or without cortical breakthrough^9^ (Figure 1). While many of these features can be confirmed on X-ray, computed tomography (CT) is the imaging modality of choice when diagnosing FD lesions.^1,8–10^ Lesions that may have radiographic similarities to FD include ossifying fibromas, osteofibrous dysplasia, giant cell tumors, Langerhans cell histiocytosis, and aneurysmal bone cysts.^8^ In cases for which the diagnosis remains uncertain based on radiographic or clinical features, or when there is concern for underlying malignancy, a biopsy may be performed for histologic and genetic analysis testing for causative *GNAS* mutations. Several look-alikes of FD, including osteofibrous lesions^12^ and fibrocartilaginous dysplasia, which has recently been classified as a form of FD,^13^ may be more accurately diagnosed on genetic testing of the lesion.

**Figure 2.**
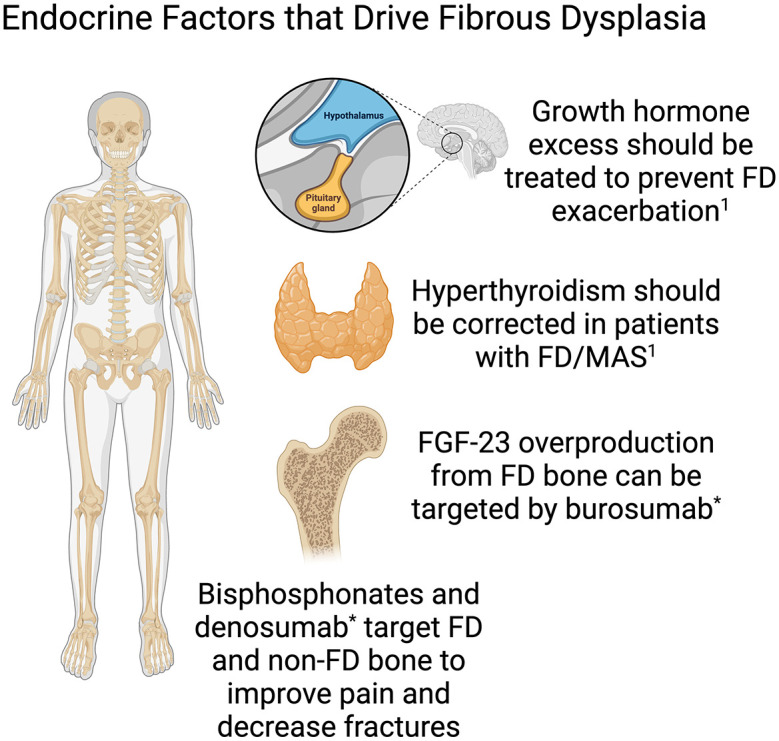
Hormone signaling in multiple organ systems have been found to impact FD lesion growth. Therapeutic strategies targeting these systems may be helpful in controlling FD. Source: Created with Biorender. ^1^Javaid et al.^8^ *Investigational use only not FDA approved for FD/MAS. FD, fibrous dysplasia; MAS, McCune–Albright Syndrome.

Careful staging of FD is necessary at the time of diagnosis to identify other potential sites of skeletal involvement. Nuclear medicine studies, such as ^99^mTc-MDP bone scans or X-ray skeletal surveys, can help identify the distribution of FD lesions. Monostotic FD is the most common form of FD, accounting for 70%–80% of all cases.^8^ Polyostotic FD is often associated with syndromic conditions such as McCune–Albright or Mazabraud syndromes, the management of which is outside the scope of this review but are covered in the treatment guidelines.^8^ All patients with FD should be screened for endocrinopathies that are associated with MAS, as this can have a significant impact on their bone health and disease course^8^ (Figure 2).

In summary, the initial diagnosis of FD is heavily dependent on the imaging characteristics, particularly obtained by CT. Staging of FD distribution can be beneficial for understanding the distribution of lesions. Patients with FD should be screened for endocrinopathies and skin lesions that may be characteristic of MAS.

## New methods for improving the genetic diagnosis of FD

Advances in genetic testing are transforming the diagnostic landscape for FD. New genetic strategies offer more precise and reliable identification of the genetic changes characteristic of this mosaic condition. Historically, the mosaic nature of FD has posed significant challenges for genetic diagnosis, particularly when relying on traditional blood specimen analyses. However, recent innovations are overcoming these obstacles, providing new avenues for accurate and early detection.

Germline genetic testing of blood or saliva samples for the *GNAS* mutation is not recommended because FD/MAS is caused by somatic mutations, and these tissue components do not typically contain high levels of the *GNAS* mutation to allow for reliable detection. One promising development is the use of cell-free DNA (cfDNA) for genetic testing in FD/MAS patients. This technique allows for the identification of genetic variants associated with FD from peripheral blood samples, circumventing the issues caused by mosaicism.^14^ The detection of cfDNA offers a minimally invasive method to obtain genetic information, enhancing the feasibility of diagnosis when a tissue sample cannot be directly obtained. This method has yet to be translated into the clinic for the diagnosis of FD/MAS, and it remains unknown whether the disease burden (i.e., number of tissues affected) of FD/MAS impacts the sensitivity of the assay in humans.

More recently, there has been an increasing interest in direct genetic testing of FD lesion samples to confirm the presence of a somatic activating *GNAS* mutation. Numerous laboratories and institutions now provide this service, including tests such as the University of California, San Francisco (UCSF) UCSF500 diagnostic panel (https://genomics.ucsf.edu/UCSF500, accessed October 18, 2024). This panel uses next-generation sequencing to detect mutations in over 529 oncologic genes and select introns of 47 genes from lesional tissue samples and can be paired with blood or non-lesional tissue to differentiate somatic from germline mutations. Although this has been used primarily in cancer patients, our experience at UCSF is that the UCSF500 can be helpful for molecularly identifying FD caused by known activating *GNAS* mutations, especially in situations where a lesion may have nonclassical clinical or radiologic features. This can be helpful to provide a better sense of the clinical trajectory and provide reassurance to the patient regarding the diagnosis.

Although not currently used in the clinical setting, single-cell RNA sequencing (scRNAseq) has emerged as a powerful tool to dissect the cellular heterogeneity within FD lesions and has been applied to FD mouse models.^15^ By analyzing individual cells from FD tissue, scRNAseq provides detailed insights into the gene expression profiles and cellular composition specific to FD, revealing differences compared to healthy tissue. This technology holds promise for the identification of distinct molecular signatures, which can be crucial for diagnosing FD and differentiating it from other similar conditions.

Moreover, recent studies have leveraged advanced imaging techniques combined with genetic analysis to improve diagnostic accuracy. Detailed phenotyping and medical image analysis have been employed to identify fibrous tissue patterns consistent with FD bone lesions, offering a noninvasive diagnostic tool.^16,17^ The application of novel techniques such as radiomics (quantitative analysis of imaging)^18–20^ and neural network/deep learning systems^21,22^ will also help improve the specificity of diagnosing FD. These methods promise to enhance the specificity of FD diagnosis, making it easier to distinguish FD from other bone disorders.

In summary, genetic testing of blood or saliva does not have high sensitivity for the *GNAS* mutation that causes FD/MAS because of the tissue mosaicism of the disease. However, lesion-specific genetics can be very useful for distinguishing FD from other types of fibro-osseous lesions, particularly if atypical features are present. Research is ongoing to improve the diagnostic specificity by combining phenotype and genotype data.

## Emerging pharmacologic strategies for FD

Over the past 10 years, there has been a great deal of new knowledge regarding the pathophysiology of FD and the key role of hormones as drivers of the FD bone formation process. This has led to the identification of emerging pharmacologic strategies that may have benefits for managing FD (Figure 2). Comprehensive guidelines outlining the pharmacologic management of FD were published in 2019 and remain the standard when caring for patients with FD/MAS^8^ and are being updated. Currently, there are no FDA-approved treatments specific for FD, and the mainstays of therapy are to control pain; ensure that abnormalities in mineral metabolism such as calcium, vitamin D, and phosphorus are corrected; and to prevent skeletal and extra-skeletal disease complications that may impact FD bone growth, including acromegaly or IGF-1 excess, hyperthyroidism, and FGF23-mediated hypophosphatemia. Pharmacologic and nonpharmacologic options for pain control, including the use of bisphosphonates, are discussed in more detail below. Bisphosphonates have been a mainstay of therapy, but clinical trials have not shown that bisphosphonates have any effect on FD lesion regression, and their utility has been largely limited to pain control in select individuals.^23^ The pharmacologic agents described below are not FDA approved for FD, and the use of these medications should be done with the benefit of consulting experts in FD/MAS, as they have known side effects and FD-specific risks that may limit their use.

### Denosumab

Denosumab, a human monoclonal antibody targeting the receptor activator of nuclear kappa factor B ligand (RANKL), has recently emerged as a potential treatment for adults and children with FD of the bone, although it is not currently FDA approved for this condition. Historically, the use of denosumab has been limited to off-label use in individual patients, many of whom showed an improvement in pain and a reduction in FD lesion size.^24^ Significant rebound bone turnover is seen infrequently—sometimes with severe hypercalcemia.^25^

To better assess the efficacy of denosumab, an observational study of 12 patients with FD or FD/MAS evaluated the effect of denosumab in patients who had a failed biochemical or analgesic response to long-term bisphosphonates.^26^ The patients were treated with denosumab 60 mg subcutaneously every 3 months for a median duration of 15.5 months. Ten patients experienced a reduction in pain, with six patients reporting complete resolution of their pain, and all patients demonstrated a decrease in bone turnover markers. This study did not assess for rebound after denosumab discontinuation.

To help address the question of rebound bone turnover after denosumab cessation, an observational study was conducted in which 37 patients with FD, many of whom had previously received bisphosphonates with no improvement, were subsequently treated with denosumab.^27^ All patients were followed up for approximately 3 years, and during that time, there was no evidence of lesion progression, fractures, or pain flares. No post-denosumab hypercalcemia was seen, but alkaline phosphatase (ALP) increased 6 months after treatment cessation and returned to baseline by 18 months; Type 1 N-terminal propeptide (P1NP) and C-terminal telopeptide (CTX) increased above pretreatment levels in 44% and 89% of patients, respectively. The patients with the highest CTX elevations prior to initiating denosumab had the highest rebound. Sixteen patients stopped the treatment after a median of 1.6 years because it did not have a significant impact on pain. These results showed a positive effect on pain in some, but not all patients, and a period of asymptomatic rebound after withdrawal without evidence of lesion growth.^27^

A recent phase II open-label study was conducted at the NIH Clinical Center (NCT03571191) to determine the effect of high-dose denosumab on FD lesion activity and bone turnover after cessation.^28^ Eight women were treated with denosumab 120 mg subcutaneously every 4 weeks for 6 months, with loading doses on weeks 2 and 3 of treatment. They were given zoledronic acid 5 mg IV 4 weeks after the final dose of denosumab and were monitored for 8 months after therapy discontinuation. At the 6-month mark, patients treated with denosumab showed significant reductions in P1NP and CTX, reduced lesion activity on NaF positron emission tomography-computed tomography (PET-CT), and a decrease in cell proliferation and lesional bone formation on bone biopsy.^29^ One patient developed severe hypercalcemia (23 mg/dL) 12 weeks after denosumab was discontinued and 8 weeks after zoledronic acid was administered, although this patient had the highest level of bone turnover at baseline and after discontinuation compared to the other seven patients. This study highlights the potential benefits of denosumab in preventing lesion growth and reducing pain and complications of FD. However, this must be carefully balanced with the risk of rebound bone turnover effects that can occur upon cessation. A clinical trial investigating the use of denosumab in children with FD is currently ongoing (NCT05419050).

A retrospective multicenter study across 6 rheumatology centers in France evaluated 13 adult patients with FD or FD/MAS who had received prior exposure to bisphosphonates for approximately 4.7 years, followed by denosumab.^30^ Denosumab was associated with a significant improvement in pain and a 30% decrease in lesional volume on MRI. Denosumab was well tolerated with no signs of hypercalcemia after discontinuation.

Although denosumab holds significant promise for the management of FD bone lesions, recent data from osteoporosis clinical trials have shown that discontinuation of denosumab can have significant consequences on bone health in this population, including a rapid increase in bone turnover and increased fracture risk upon treatment cessation.^31^ For this reason, discontinuation of denosumab is not recommended. If denosumab must be discontinued, administration of a bisphosphonate—either alendronate, or 1 or more doses of zoledronic acid—6 months after the last denosumab dose—is currently recommended.^32^ However, this may not fully mitigate the rebound effects, particularly in patients who have been on denosumab for a longer duration or higher dose, and remains an area of active investigation in the osteoporosis population, with no systematic data in patients with FD. The potential for serious rebound effects in FD patients must be carefully considered before this medication is prescribed off-label for patients with FD, and the authors do not recommend initiating denosumab without consulting with an expert in FD.

In summary, denosumab shows promising benefits for FD bone lesion regression and pain but appears to have significant potential adverse effects when discontinued. Denosumab is also not currently FDA approved for use in FD, and research into its use in FD is ongoing.

### Phosphate wasting assessment and management

FD lesions have been shown to be high producers of FGF23, leading to FGF23-mediated renal phosphate wasting.^33^ Phosphate wasting is an important predictor of FD disease as well as future fracture risk, including in areas of the skeleton not affected by FD.

The most reliable assessment of phosphate equilibrium is performed by measuring serum and urine phosphate and creatinine after an overnight fast to avoid diet-related effects on phosphate levels and after discontinuing phosphate supplements for at least 1 day. The tubular reabsorption of phosphate (TmP/GFR) can then be calculated and compared to age-based reference ranges. Hyperparathyroidism and renal tubular acidosis should be excluded by biochemical assessment as well. FGF23 levels can be measured as additional confirmation, after at least a 1-week pause from vitamin D, calcitriol, or phosphate-containing supplements. Patients should be monitored carefully while off supplements.

The mainstay of treatment for FGF23-mediated phosphate loss is currently vitamin D analogs, such as calcitriol or alfacalcidol. Periodic monitoring of urine calcium levels is necessary to risk-stratify patients for nephrolithiasis during the treatment course.^8^ Phosphate supplements may also be necessary in the short term, but they must be balanced with the long-term risk of chronic parathyroid stimulation and hyperplasia. Parathyroid hyperplasia may preclude further pharmacologic treatment with either class of medications, so parathyroid hormone-level monitoring is necessary while patients are being supplemented with phosphate.

In summary, FD is associated with FGF23 production and the development of hypophosphatemia. Phosphate management has significant clinical benefits for patients with FD/MAS.

### Burosumab

As a result of the complications related to phosphate metabolism in FD/MAS, there has been great interest in new medications that can target the excess FGF23 production seen in FD.^33–35^ Burosumab is a monoclonal antibody targeting FGF23 and is FDA approved for X-linked hypophosphatemic rickets and tumor-induced osteomalacia.^36,37^ FD lesions produce FGF23,^33^ which can cause hypophosphatemia and subsequent hypophosphatemic bone pain and rickets. These complications can be challenging to treat and may lead to persistent bone pain. Additionally, untreated hypophosphatemia is a contraindication to medications such as bisphosphonates or denosumab. A phase II, open-label study, Burosumab for Fibroblast Growth Factor-23 Mediated Hypophosphatemia in Fibrous Dysplasia (NCT05509595), is currently ongoing for adult and pediatric patients.^38^

In summary, targeting excess FGF23 that may be present in patients with FD may be a useful adjunct for managing calcium/phosphate levels, as well as the bone disease in FD. Further studies on the use of burosumab in FD are underway.

## Management of FD/MAS-associated pain

FD/MAS is often associated with deformity, fractures, and dysfunctional chronic pain which significantly impact patients’ quality of life.^39,40^ Pain is reported by 46%–67% of patients with FD,^41–43^ and is more common in adults than children (81% vs 49%).^42^ Mainly described as “bone pain,” chronic FD-associated pain is complex and often challenging to manage. Based on the characteristics and nature of pain generators, pain is classified as nociceptive pain, inflammatory pain, and pathological pain, which includes both neuropathic pain associated with nerve damage^44^ and nociplastic pain, which has no clear evidence of tissue injury.^45^ Exacerbated by acute injury, nociceptive pain is commonly expected in patients with FD. However, a recent retrospective registry study identified neuropathic-like pain in 31% of patients with FD.^46^

The mechanism of chronic FD-associated pain remains poorly understood and is likely multifactorial. As nociceptive nerves, mainly CGRP^+^, are widely distributed in the periosteum and marrow cavity of bone,^47,48^ tissue destructive injury during the expansion of FD bone lesions may induce nociceptive nerve sprouting and contribute to bone pain.^42^ This hypothesis is supported by some encouraging results from antiresorptive therapies targeting osteoclastogenesis and bone resorption,^49^ which led to pain reduction in some patients.^50^

To manage pain, it is important for providers to identify any treatable systemic pain generators before focusing on symptomatic control. As FD bone is more susceptible to fractures,^51^ which can occur without patients’ awareness, new onset of severe acute pain or acute pain without improvement should be flagged for further evaluation, including surgical consultation. Surgical intervention may be necessary for correcting severe pathological fractures or deformities and often leads to pain reduction in patients with FD.^8^

**Figure 3.**
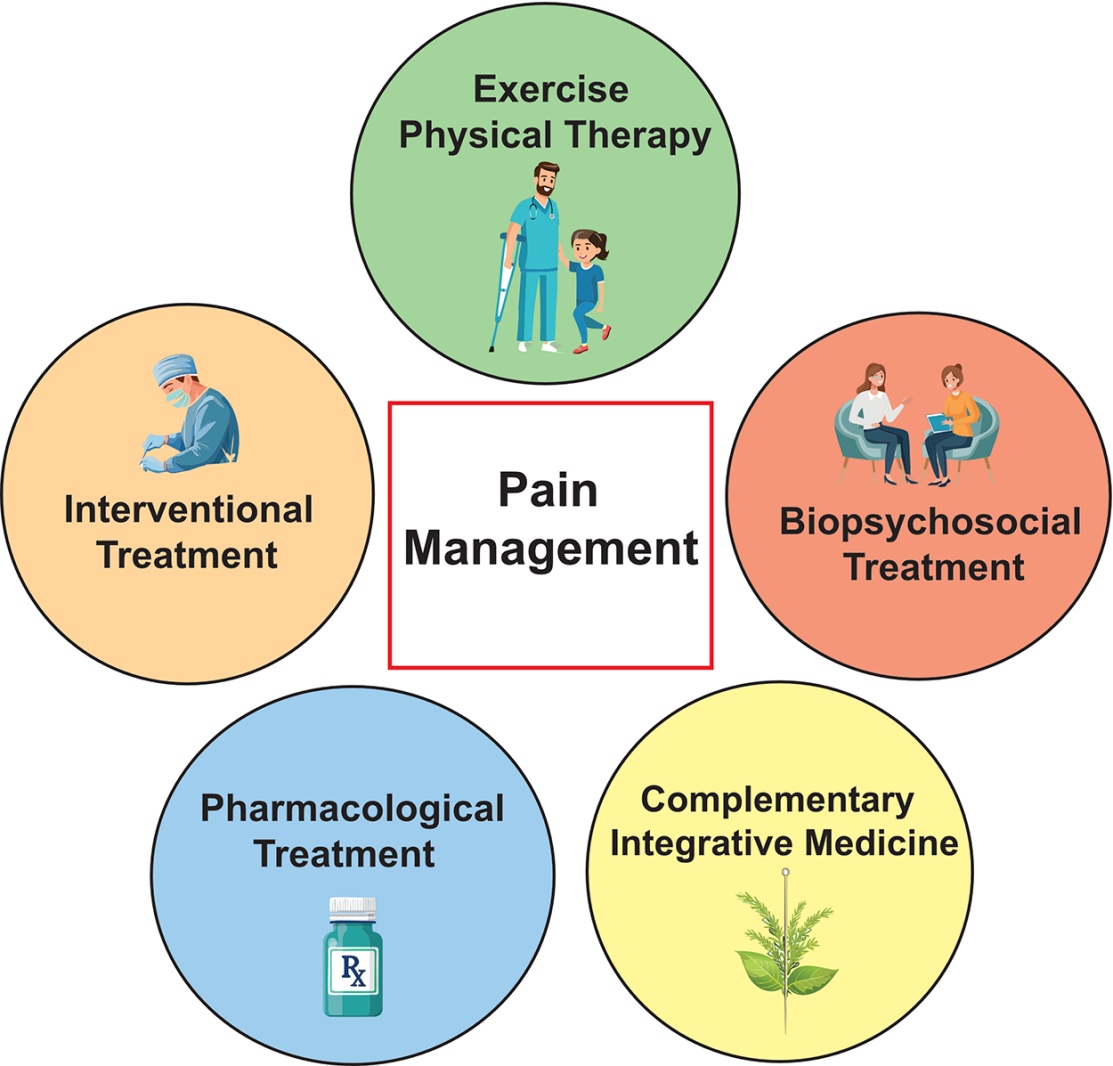
The schematic illustration of the pathway of multidisciplinary pain management. Source: Cartoon images were generated by Adobe Illustrator Generate Vector (beta).

With an increasing recognition that acute pain medicines can become less effective for chronic pain in part due to the changes in neural circuitry,^52^ multidisciplinary pain management has become the standard care.^53,54^ Following the existing treatment guidelines for the management of FD-associated pain,^8,55^ we propose multidisciplinary care from a team of FD specialists, surgeons, pain specialists, nurses, physical therapists, and psychologists, with a special interest in FD. If chronic pain is not adequately relieved with pharmacotherapy, physical therapy (PT), interventional procedures such as pain injections and surgery, biopsychosocial therapy, and complementary/integrative medicine can be considered^53,54^ (Figure 3). Importantly, the outcome of multidisciplinary care in patients with FD/MAS has been assessed.^56^ In this observational study, new patients enrolled in the pathway reported clinically significant improvements in both physical function (*p* = 0.020) and maximal pain reduction (*p* = 0.038) during 1 year of follow-up. Moreover, emotional well-being was more improved in the existing patients (*p* < 0.001).

In summary, FD is associated with significant pain and discomfort, and this has a major impact on the quality of life for patients with FD. A multimodal approach to pain management is extremely beneficial for patients with FD/MAS.

### Education on FD pain management

Lack of resources and knowledge is a major barrier to managing chronic FD-associated pain effectively and collaboratively. More educational efforts are required to inspire more health care providers to be committed to improving quality, equity, and access to multidisciplinary care.^57,58^ Healthcare professionals will benefit from education and training to become more comfortable managing symptomatic pain safely. It is also important to increase community awareness of FD in patients and caregivers through active outreach and teaching via conferences, newsletters, social media, and helplines in collaboration with support groups for patients with FD.^54,57,59^

Although this review focuses on the medical management of FD, it is important for patients to be aware that surgical intervention may be necessary for correcting severe pathological fractures or deformities. In these extreme situations, surgical intervention can often lead to pain reduction in patients with FD while stabilizing bone biomechanics.^8^ Close coordination with a pain specialist team for potential interventional pain procedures may also be helpful for improving pain relief and quality of life.^53,54^

In summary, patients with FD-related pain can benefit significantly from counseling and education in different pain management strategies.

### Nonpharmacologic pain management

Due to its low risk, nonpharmacologic treatment with superficial heat or cold, massage, acupuncture, exercise, PT, and psychological therapy^53,54^ should be initially selected for conservative management of both acute and chronic FD pain.

As the most commonly recommended nonpharmacologic treatment to reduce pain and regain functionality,^53,54^ restorative therapy such as exercise and PT is often considered as a first-line approach to manage many chronic pain conditions including fibromyalgia,^60^ low back pain,^61,62^ and migraine.^63^ Although it has been recommended to help preserve ambulation ability in patients with FD by improving hip strength and range of motion,^64^ the efficacy of PT and exercise in functional improvement in patients with FD has not been well studied.

Physical activity and mental health are intricately linked. Depression, anxiety, and stress are common comorbidities in patients with chronic pain and can negatively affect the outcome of treatment. Cognitive behavioral therapy is an evidence-based treatment for the affective component of pain by increasing coping skills to reduce anxiety and stress^65^ and can improve patients’ overall function.^66–68^ Therefore, offering psychological support can effectively address affective pain and improve overall well-being.

In summary, patients with FD benefit significantly from nonpharmacologic modes of pain management. This is an important strategy to complement pharmacologic approaches for FD pain care.

### Pharmacological pain management

The mainstay of current pharmacological pain treatment is to employ a multimodal approach to target different pain pathways. Acetaminophen and nonsteroidal anti-inflammatory drugs (NSAIDs) are often used first. NSAIDs block the production of prostaglandins by inhibiting cyclooxygenase (COX).^69^ However, NSAIDs must be used with caution due to well-known side effects such as kidney damage, increased bleeding by affecting platelet function, and potential cardiovascular risks.^70^ Celecoxib, a COX2 inhibitor, is known to have fewer gastrointestinal side effects than nonselective inhibitors such as naproxen or ibuprofen.^71^ At moderate doses (100 mg PO BID), its cardiovascular safety was found to be noninferior to ibuprofen (600 mg PO TID) or naproxen (375 mg PO BID).^71^ Interestingly, it was reported that celecoxibinhibited FD cell proliferation *in vitro* and reduced FD-like lesion progression *in vivo*.^72^ To reduce the risk of systemic adverse effects, topical NSAIDs have been developed, and the efficacy and safety have been supported by several randomized, double-blind, placebo-controlled (RCT) studies.^73^

If neuropathic pain features are noted, anticonvulsants and antidepressants can be trialed.^53,54^ Due to fewer adverse effects, the gabapentinoids, a class of anticonvulsant medications, are most commonly used. The doses should be escalated slowly while monitoring side effects such as sedation. Clinicians should be aware that the dose needs to be reduced in patients with end-stage renal disease. Tricyclic antidepressants such as nortriptyline and amitriptyline, and serotonin norepinephrine reuptake inhibitors such as duloxetine and venlafaxine are also commonly used.^53,54^

Opioid therapy is no longer the first-line option for pain and is generally reserved for severe pain that is recalcitrant to conventional therapy. To reduce the risks of chronic opioid use, buprenorphine has been increasingly used in treating chronic pain. Emerging evidence supports that it has similar analgesic efficacy to full mu-receptor agonists.^74,75^ As a partial mu agonist and kappa antagonist,^76^ buprenorphine usually does not have clinically significant respiratory depression.^77^ However, it is highly recommended to seek guidance from pain specialists for appropriate use.

Bisphosphonates may provide relief from FD-related pain in patients with moderate to severe pain, although there is no supportive evidence that they slow the progression of FD or decrease the lesion size.^23^ Zoledronic acid or pamidronate, both administered intravenously, are the bisphosphonates of choice and may improve pain in >50% of adults with FD.^78^ Bisphosphonate use for up to 6 years was associated with complete clinical and biochemical improvement in 24 out of 30 patients with polyostotic FD but was less effective in patients with MAS, perhaps reflective of the greater underlying disease burden.^79^ We support the approach and dosing outlined by the FD/MAS International Consortium Consensus Guidelines when initiating IV bisphosphonates for pain,^8^ which suggests an adult regimen of zoledronic acid 5 mg intravenously monthly for up to three doses and then a yearly maintenance dose, or pamidronate 90 mg/day intravenously for 2 consecutive days every 6 months (up to two cycles). At this time, there is insufficient evidence to suggest that a particular intravenous bisphosphonate is superior to another. Risks from long-term bisphosphonate use should also be considered. A randomized-controlled clinical trial using alendronate, an oral bisphosphonate, administered at doses of 40 mg orally per day, did not improve FD-related pain^80^; therefore, oral bisphosphonates should not be used as the first choice for FD pain management. As discussed previously, denosumab is an emerging therapy that may help reduce FD lesion pain and reduce lesion growth, while burosumab is being investigated for its potential role in improving pain related to FGF23-mediated hypophosphatemia and osteomalacia.

In summary, pain management for FD may require medications. Traditional pain medications can be very helpful, as well as the addition of bisphosphonates, which can help reduce pain but do not have a significant impact on lesion progression. Detailed studies for the use of denosumab or burosumab in FD pain management are underway.

### Cannabinoids in pain management

Cannabis and selective cannabinoids containing tetrahydrocannabinol and/or cannabidiol have become increasingly popular for managing many diseases including chronic pain. Although the antinociceptive effect of cannabinoids has been well demonstrated in preclinical studies, translating this into clinical therapies remains challenging with conflicting evidence. A recent systematic review of RCTs did not find sufficient evidence to either support or refute claims of efficacy and safety for cannabinoids and cannabis in pain management.^81^ Another systemic review concluded that use of selective cannabinoids in patients with chronic neuropathic pain was associated with improvements in quality of life and sleep.^82^ However, there are serious concerns regarding potential impact on neurological development in children, cannabis dependence, and drug interactions with anesthetics in regular users undergoing surgery.^83^ As the evidence base is weak, cannabinoids/cannabis should be used with caution, and patients are encouraged to discuss with their providers first.

In summary, as the underlying causes of FD-associated pain are multifactorial, we recommend individualizing pain management to identify contributing pain factors through multidisciplinary care; employ multimodal therapy; and promote education for patients, their families, and healthcare providers.

## Skeletal stability assessment and management

Assessment of the skeletal system for FD patients also involves obtaining a comprehensive fracture history with the level and type of fracture and subsequent orthopedic procedures. Bone turnover markers such as ALP, PINP, and CTX may also be of assistance and correlates with FD-related fracture risk.^51,84^

There are no specific guideline recommendations regarding the use of Dual Energy X-ray Absorptiometry (DXA) for bone density measurements of FD lesions. However, bone mineral density (BMD) screening for non-FD bone may be reasonable for patients with traditional osteoporosis risk factors or severe renal phosphate wasting for further risk stratification and management. DXA analysis of FD lesions will be inaccurate due to the changes in bone density and geometry from the lesions.

If the patient has a history of fractures or exhibits red flag signs of imminent fracture such as night pain or severe weight-bearing pain, a multidisciplinary approach involving an orthopedic surgeon with expertise in FD is necessary.^8^ Scoliosis, limb deformity, and leg length discrepancy may be treatable with orthotics or corrective surgery. As in other cases of pathologic fractures, external fixation is only seen as a temporary treatment while waiting for a definitive implant. Internal fixation is preferably done using a custom-made titanium intramedullary nail or other custom hardware, but occasionally plate fixation can be considered. Staged procedures may be necessary as FD lesions are vascular, and there may be significant blood loss. Whether perioperative bisphosphonates in the context of fracture improve outcomes is yet unclear. A recent meta-analysis^50^ showed that antiresorptive drugs as a whole have been shown to reduce bone turnover marker levels and increase BMD in both FD and non-FD bone modestly, but bisphosphonates do not significantly reduce FD lesion size or slow the progression of disease.

Finally, although progression of an FD lesion to osteosarcoma is rare and described primarily in case reports,^85–87^ it is important for patients and clinicians to be aware of unusually rapidly growing lesions or significant changes in pain patterns that may indicate malignant transformation.

In summary, patients with FD should be followed for bone health, particularly since FD bone lesions are at an increased risk of fracturing. Surgical stabilization may be beneficial for pain management and to decrease skeletal deformities.

## Special considerations for craniofacial and dental FD

Craniofacial fibrous dysplasia (CFD) is one of the most common locations for the disease to develop.^3,43^ Signs and symptoms can include facial asymmetry, pain, and functional impairments, including vision and hearing loss. CFD typically first presents in childhood but can be incidentally discovered later in life as part of a workup for headaches, hearing loss, or after head trauma. Surgical intervention is challenging due to risks of regrowth and complications, and is generally reserved for cases with significant functional or esthetic compromise.^3,8^

The most important management goals of craniofacial FD include prevention of functional loss, such as hearing, vision, and mastication, as well as prevention and improvement of any deformities. A multidisciplinary care team involving craniofacial surgery, ENT, and ophthalmology services is recommended; thin cut CT (1 mm) scans to characterize and monitor lesions are recommended approximately every 2 years in children and no more than every 5 years in asymptomatic adults.^8^ Craniofacial FD is particularly challenging due to the high variability of lesion characteristics on imaging (Figure 1), and interventions are often challenging due to the location and proximity to vital craniofacial structures.^3,8^ The use of lesion-specific tissue genetics can be very helpful if a biopsy is needed to confirm the diagnosis of FD; however, routine biopsies are generally not recommended.^8^ Monitoring for skull base involvement and associated neurologic sequelae is also crucial, as is routine ophthalmologic evaluation for patients with orbital involvement and hearing testing for patients with temporal bone involvement.

Patients with craniofacial FD can exhibit various dental anomalies due to involvement of the mandible and maxilla.^88–90^ These anomalies include dental displacement, malocclusion, and facial asymmetry. Teeth may become crowded, abnormally spaced, or rotated, and can show splaying of roots around FD lesions. Specific dental phenotypes reported include oligodontia, enamel hypoplasia, dentin dysplasia, taurodontic pulp, odontoma, tooth displacement, malocclusion, and high rates of dental caries. Panoramic radiographs and intraoral radiographs are recommended for thorough assessment and management of dental anomalies, and to monitor the risk factors for osteonecrosis of the jaw, particularly in the setting of bisphosphonate or denosumab treatment.^8^ Whether *GNAS* mutations can occur in the cells related to tooth development, such as ameloblasts or odontoblasts, and how this might affect the dental phenotypes in FD, are still largely unknown.

## Summary/conclusion

Our understanding of the disease pathogenesis of FD/MAS continues to improve and helps guide our management strategies for this debilitating condition. Updated treatment guidelines are planned for 2025. Many areas of research still remain, including identifying molecular targets and strategies that would directly inhibit and potentially reverse FD bone growth, as has been seen in some mouse models of FD.^49,91^ In addition, identifying biomarkers of disease progression, as well as understanding the role of genetic mutation burden in FD lesion progression, remain areas of great interest in research. Finally, our understanding of what drives pain in FD remains rudimentary. A better understanding of these pathologic processes would help improve pain management in patients with FD, with potentially significant impact on their quality of life.

## Declarations

**Ethics approval and consent to participate:** Not applicable.

**Consent for publication:** Consent was received for the publication of the patient images.

**Competing interests:** E.C.H. serves in an unpaid capacity on the Medical Registry Advisory Board of the International Fibrodysplasia Ossificans Progressiva Association; the Fibrous Dysplasia Foundation Medical Advisory Board and Scientific Advisory Board; and the International Clinical Council on FOP. E.C.H. receives clinical trials support through his institution from Clementia Pharmaceuticals, an Ipsen Company; Ipsen Pharmaceuticals; Ascendis Bio; and a¯shibio. K.L.W. receives clinical trials support through her institution from Ascendis Bio and a¯shibio. J.P. and X.Y. have no disclosures to declare.

**Availability of data and materials:** Not applicable.
